# Diagnostic Utility and Impact on Clinical Decision Making of Focused Assessment With Sonography for HIV-Associated Tuberculosis in Malawi: A Prospective Cohort Study

**DOI:** 10.9745/GHSP-D-19-00251

**Published:** 2020-03-30

**Authors:** Daniel Kahn, Kara-Lee Pool, Linna Phiri, Florence Chibwana, Kristin Schwab, Levison Longwe, Ben Allan Banda, Khumbo Gama, Mayamiko Chimombo, Chifundo Chipungu, Jonathan Grotts, Alan Schooley, Risa M. Hoffman

**Affiliations:** a Department of Internal Medicine, David Geffen School of Medicine, University of California at Los Angeles, Los Angeles, CA, USA.; b Department of Radiology, David Geffen School of Medicine, University of California at Los Angeles, Los Angeles, CA, USA.; c Partners in Hope, Lilongwe, Malawi.; d Department of Medicine, Division of Pulmonology, David Geffen School of Medicine, University of California at Los Angeles, Los Angeles, CA, USA.; e Department of Medicine Statistics Core, David Geffen School of Medicine, University of California at Los Angeles, Los Angeles, CA, USA.; f Department of Medicine, Division of Infectious Disease, David Geffen School of Medicine, University of California at Los Angeles, Los Angeles, CA, USA.

## Abstract

**Background:**

The focused assessment with sonography for HIV-associated tuberculosis (TB) (FASH) ultrasound protocol has been increasingly used to help clinicians diagnose TB. We sought to quantify the diagnostic utility of FASH for TB among individuals with HIV in Malawi.

**Methods:**

Between March 2016 and August 2017, 210 adults with HIV who had 2 or more signs and symptoms that were concerning for TB (fever, cough, night sweats, weight loss) were enrolled from a public HIV clinic in Lilongwe, Malawi. The treating clinicians conducted a history, physical exam, FASH protocol, and additional TB evaluation (laboratory diagnostics and chest radiography) on all participants. The clinician made a final treatment decision based on all available information. At the 6-month follow-up visit, we categorized participants based on clinical outcomes and diagnostic tests as having probable/confirmed TB or unlikely TB; association of FASH with probable/confirmed TB was calculated using Fisher's exact tests. The impact of FASH on empiric TB treatment was determined by asking the clinicians prospectively about whether they would start treatment at 2 time points in the baseline visit: (1) after the initial history and physical exam; and (2) after history, physical exam, and FASH protocol.

**Results:**

A total of 181 participants underwent final analysis, of whom 56 were categorized as probable/confirmed TB and 125 were categorized as unlikely TB. The FASH protocol was positive in 71% (40/56) of participants with probable/confirmed TB compared to 24% (30/125) of participants with unlikely TB (odds ratio=7.9, 95% confidence interval=3.9,16.1; *P*<.001). Among those classified as confirmed/probable TB, FASH increased the likelihood of empiric TB treatment before obtaining any other diagnostic studies from 9% (5/56) to 46% (26/56) at the point-of-care. For those classified as unlikely TB, FASH increased the likelihood of empiric treatment from 2% to 4%.

**Conclusion:**

In the setting of HIV coinfection in Malawi, FASH can be a helpful tool that augments the clinician's ability to make a timely diagnosis of TB.

## Abstract (precis)

Among patients with HIV and with probable/confirmed TB, using the focused assessment with sonography for HIV-associated TB (FASH) protocol led to a 5-fold increase in the clinician's decision to initiate TB treatment on that day. FASH is a supplementary tool that can help clinicians diagnose patients with HIV-associated TB at the point-of-care and reduce delays in their treatment, particularly when access to other diagnostics is limited or unavailable.

## INTRODUCTION

The risk of developing active tuberculosis (TB) is 20–37 times higher in people living with HIV than in people who do not have HIV.^1^ This risk is compounded by difficulty in diagnosing TB in individuals who have HIV, as they more commonly present with atypical radiographic findings, smear-negative TB, and disseminated extrapulmonary manifestations.^2^ As a result, individuals with HIV and TB have a higher mortality rate, likely due to diagnostic uncertainty that leads to delays in therapy.^3^ Gold standard diagnostics, such as TB culture, are often unavailable in regions with the highest burden of TB and HIV. Even when these diagnostics are available, the results can take up to 6 weeks to return, causing delays in diagnosis and treatment. A recent autopsy study investigating patients with HIV who died in an inpatient ward in South Africa found that TB was implicated in two-thirds of those deaths and one-third of those cases were not diagnosed and treated at the time of death.^4^

To reduce TB morbidity and mortality, decrease transmissibility, and gain control of the epidemic, improved point-of-care diagnostics in resource-limited settings are needed. Ultrasound is one such diagnostic modality. Point-of-care ultrasound (POCUS) provides the clinician with immediate feedback to make informed decisions that can avoid delays in treatment. One POCUS protocol, focused assessment with sonography for HIV-associated TB (FASH), has been developed to improve the diagnosis of extrapulmonary TB in patients with HIV by evaluating for pericardial fluid, pleural fluid, ascites, abdominal lymphadenopathy, and hepatic and splenic hypoechoic focal lesions.^5^ These sonographic findings of HIV-associated extrapulmonary TB have been described in various settings and populations.^6^^–^^13^ The FASH protocol has been shown to detect TB that would be missed based on other available diagnostics.^14^^,^^15^ In many resource-limited settings, POCUS is more available than regular radiology services (due to availability of low-cost, small, rechargeable ultrasound devices) and point-of-care lab diagnostics, which require equipment, a regular electricity source, a supply chain for reagents, and trained technicians. FASH can be taught to midlevel health workers and has been successfully implemented in resource-limited settings.^5^^,^^16^^–^^19^ It is increasingly plausible that rural facilities could rely only on history, physical exam, and FASH to make an initial assessment of TB.

The FASH protocol has been shown to detect TB that would be missed based on other available diagnostics.

Despite the reported benefits of using FASH for TB diagnosis, uncertainty remains about its sensitivity, specificity, and predictive value in settings like Malawi where exams are often performed by midlevel providers. It is also unclear how best to incorporate FASH into the TB diagnostic algorithm. We sought to quantify the predictive value of the FASH protocol for TB and to assess the impact on clinical decision making at the point-of-care in Malawi.

**Figure:**
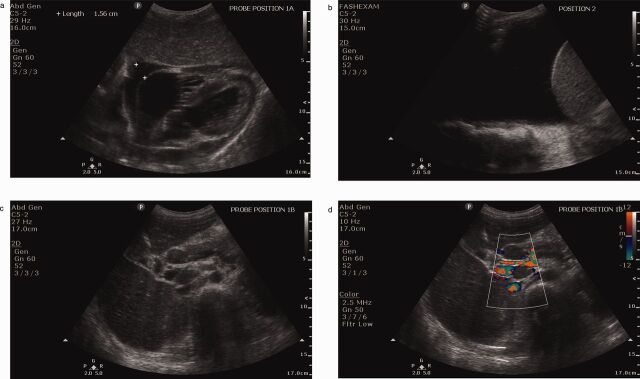
Images of Findings From Focused Assessment With Sonography of HIV-Associated Tuberculosis Protocol in Participants With Signs and Symptoms of Tuberculosis (a) Anechoic fluid surrounding the heart consistent with a moderate pericardial effusion. (b) Anechoic area superior (left) to the diaphragm and liver consistent with a large right pleural effusion. (c) Isoechoic nodules consistent with peri-portal and para-aortic lymphadenopathy. (d) Color Doppler further differentiates vasculature from lymph nodes.© 2016 Daniel Kahn/UCLA

## METHOD

### Setting

We performed a prospective cohort study at Partners in Hope Medical Center in Lilongwe, Malawi, a facility that delivers free HIV care and includes an antiretroviral therapy clinic serving approximately 5,500 clients. Staff at the medical center have the following diagnostic tests available to them when evaluating clients who present with signs and symptoms of TB: complete blood count, CD4 cell count, viral load, acid fast bacteria stains, Xpert MTB/RIF to detect *Mycobacterium tuberculosis* (MTB) and resistance to the anti-TB drug rifampicin (RIF), urine lipoarabinomannan assay (LAM), and chest radiography. In 2015, the medical center acquired an ultrasound machine, and clinicians were trained in the FASH protocol. Information about this training has been previously published.^17^^,^^18^

### Population

Between March 2016 and August 2017, we enrolled 210 adults with HIV who were 18 years of age or older who presented to Partners in Hope Medical Center for care and answered “yes” to having 2 or more of the following TB symptoms: fever, cough, night sweats, weight loss. We used convenience sampling to enroll participants. If an individual met criteria based on the routine TB screening questions, and the study and clinical staff were available to perform screening, consent, and study procedures, then the participant was screened and enrolled, if eligible. Participants were eligible regardless of antiretroviral therapy status. Individuals were excluded if they were already receiving TB treatment or were pregnant.

Written informed consent was obtained from all participants, and the study was approved by the Malawi National Health Sciences Research Committee and the University of California at Los Angeles (UCLA) Institutional Review Board.

### Study Procedures

After enrollment, the treating clinician (1 of 5 clinical officers or 1 medical doctor) conducted a history and physical exam and documented on a paper study form whether they would empirically treat for TB based on the history and physical exam findings. The treating clinician then conducted the FASH protocol, which included assessment for pericardial, pleural, and ascitic fluid; abdominal lymphadenopathy; and focal liver and splenic lesions using a Philips ClearVue 650 with a C5-2 abdominal transducer (2–5 MHz). FASH was defined as positive if any single component was positive, with the exception of trace pericardial effusions (defined as pericardial effusion <0.5 cm), which are considered clinically insignificant. After completing the FASH, the same clinician documented whether they would treat for TB based on the FASH results (before conducting any labs or other studies) to mimic scenarios that may commonly occur in settings throughout Malawi that could have access to POCUS but not point-of-care labs or chest radiography.

After FASH was completed, all participants were then evaluated with sputum microscopy, sputum Xpert MTB/RIF assay, urine LAM, complete blood count, CD4 cell count, viral load, and chest radiography. Sputum and tissue cultures were not obtained as they were not readily available in Malawi.

At the end of the clinical visit, based on all available data, the treating clinician made final management decisions, including whether to treat for TB. Participants were asked to return for study follow-up visits at 2 weeks, 3 months, and 6 months after enrollment. At each follow-up visit, a study nurse evaluated the participant for ongoing TB symptoms, took vital signs and weight, and reviewed the medical record for any new diagnostic results related to TB. Long-term management of TB treatment was performed by Partners in Hope clinicians per the standard of care in Malawi.

### FASH Quality Assurance

Still images of each FASH protocol component were shared with a UCLA radiologist using an encrypted file-sharing program. This radiologist read all images within 72 hours. Discrepancies between the clinical officer and expert radiologist review were immediately communicated to the clinical team such that patient management could be adjusted as needed. Detailed methods on FASH quality assurance have been previously published.^18^

### Definitions for Analysis

**FIGURE 1:**
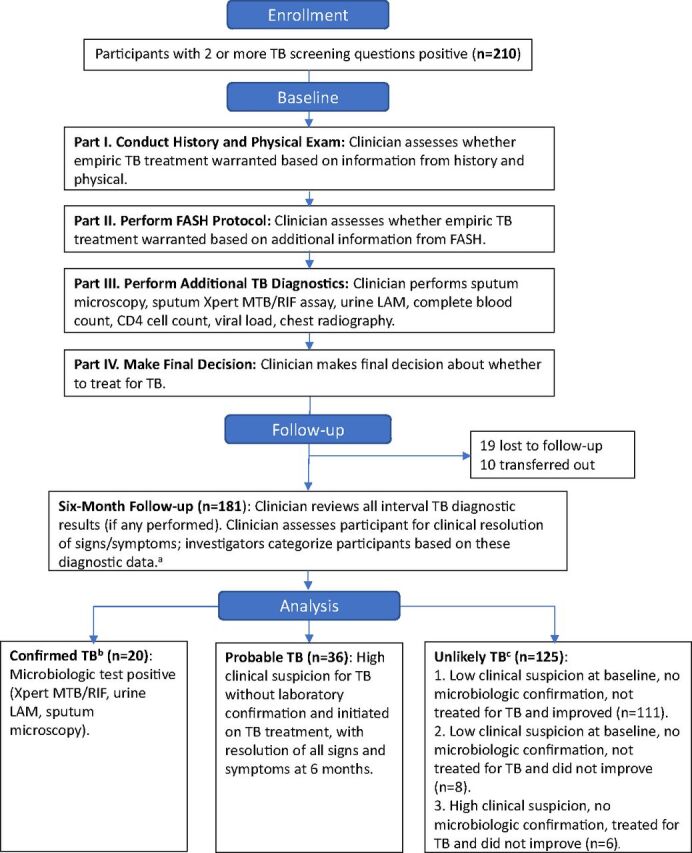
Participant Enrollment in a Prospective Cohort Study Assessing Diagnostic Utility of FASH at an Urban Medical Center, Lilongwe, Malawi, and Clinicians' Stepwise Diagnostic Evaluation and Decision Making Abbreviations: FASH, focused assessment with sonography for HIV-associated tuberculosis; LAM, lipoarabinomannan; MTB, *Mycobacterium tuberculosis*; RIF, rifampicin; TB, tuberculosis. ^a^Final categorization made by study investigators based on 6-month data and definitions above. ^b^Includes 2 deaths. ^c^Includes 12 deaths: 7 in Subgroup 2 and 5 in Subgroup 3.

After the 6-month follow-up visit, we categorized each participant into 1 of 3 diagnostic groups based on likelihood of TB: (1) confirmed TB, defined by any 1 positive microbiologic test (sputum acid fast bacteria microscopy, Xpert MTB/RIF assay, or urine LAM); (2) probable TB, defined as a clinical diagnosis of TB without laboratory confirmation and initiated on TB treatment, with resolution of all signs and symptoms at 6 months; or (3) unlikely TB, defined as no positive laboratory finding for TB and was either treated for TB and did not improve by 6 months or was not treated for TB (Figure 1).

There were no significant differences between the baseline characteristics of confirmed and probable TB groups (Supplementary Table 1); therefore, these diagnostic categories were combined into 1 group for analyses and are referred to throughout as probable/confirmed TB.

Individuals who died during the study without laboratory confirmation of TB were categorized as unlikely TB whether they were treated for TB or not based on the above definition. Participants who transferred out or were lost to follow-up were excluded from the analysis as they could not be assigned a TB category.

### Statistics

Summary statistics were performed for all baseline demographic and clinical data. We compared characteristics of participants in each diagnostic category using Wilcoxon Rank Sum tests for continuous data and Fisher's exact tests for discrete data. FASH data and associations with TB were summarized using odds ratios (OR), sensitivity, specificity, positive predictive value and negative predictive value, and positive likelihood ratio and negative likelihood ratio with 95% confidence interval (CI). A *P* value below .05 was considered statistically significant, and all tests were 2-sided. Statistical analysis was done using the R Language and Environment for Statistical Computing (R Core Team, 2019, Vienna, Austria).

## RESULTS

### Baseline Characteristics

Of the 210 participants enrolled, 19 were lost to follow-up and 10 transferred out; these 29 participants were excluded from the analysis. The 14 participants who died during the study were included in the final analysis, including 2 participants with confirmed TB, and categorized accordingly, and 12 without laboratory confirmation and were categorized as unlikely TB. Of the 181 participants included in the final analysis, we classified 56 as probable/confirmed TB (20 microbiologically confirmed and 36 probable) and 125 as unlikely TB. Compared to the unlikely TB group, those with probable/confirmed TB were more likely to have never taken antiretroviral therapy and to have had a lower CD4 count and lower body mass index (Table 1).

**TABLE 1.** Baseline Demographic and Clinical Characteristics of Participants at an Urban Medical Center, Lilongwe, Malawi, Stratified by TB Category (N=181)

	Unlikely TB (n=125)	Probable/Confirmed TB (n=56)	*P* Value
**Age**, years, median (IQR)^a^	40.0 (34.0–45.0)	39.0 (33.0–43.0)	.51
**Gender**			.42
Male, No. (%)	56.0 (44.8)	29.0 (51.7)	
Female, No. (%)	69.0 (55.2)	27.0 (48.2)	
**Baseline CD4 Count** (cells/mm^3^), median (IQR)^b^	256.0 (80.0–484.0)	117.0 (29.0–176.0)	<.001
**Viral Load** (copies/ml)^c^			.21
<1,000, No. (%)	80.0 (64.0)	28.0 (50.0)	
1,000–50,000, No. (%)	14.0 (11.2)	11.0 (19.6)	
>50,000, No. (%)	22.0 (17.6)	9.0 (16.0)	
**Baseline ART Regimen** ^d^			.001
TDF/3TC/EFV, No. (%)	70.0 (56.0)	20.0 (35.7)	
Other NNRTI-based regimen, No. (%)	17.0 (13.6)	2.0 (3.6)	
Protease inhibitor-based regimen, No. (%)	12.0 (9.6)	3.0 (5.4)	
No ART, No. (%)	25.0 (20.0)	26.0 (46.4)	
**Previous TB,**^e^ No. (%)	32.0 (25.6)	12.0 (21.4)	.58
**TB Sign/Symptom** ^f^			
Fever, No. (%)	83.0 (66.4)	42.0 (75.0)	.30
Cough, No. (%)	108.0 (86.4)	51.0 (91.1)	.47
Night sweats, No. (%)	77.0 (61.6)	37.0 (66.1)	.62
Weight loss, No. (%)	98.0 (78.4)	50.0 (89.3)	.10
**BMI,** median (IQR)	19.2 (17.5–22.9)	18.6 (16.7–19.8)	.01

Abbreviations: ART, antiretroviral therapy; BMI, body mass index; IQR, interquartile range; NNRTI, non-nucleoside reverse transcriptase inhibitor; TB, tuberculosis; TDF/3TC/EFZ, tenofovir disoproxil fumarate/lamivudine/efavirenz.

a Missing in 5 individuals.

b Missing in 4 individuals.

c Missing in 17 individuals.

d Missing in 6 individuals.

e Based on participant self report.

f Participants could have 2 or more signs/symptoms based on screening questions at study entry.

The most common positive confirmatory tests were urine LAM (13), sputum Xpert MTB/RIF (9), and sputum microscopy (7). Twenty-eight percent (50/181) of participants had an abnormal chest radiography, including 64% (36/56) of participants in the probable/confirmed TB group and 11% (14/125) of participants in the unlikely TB group. Of those who had an abnormal chest radiography, 18% (9/50) had a positive sputum (either by microscopy or Xpert).

### Comparison of Baseline FASH Findings

Seventy-one percent (40/56) of participants with probable/confirmed TB were found to have a positive FASH, compared to 24% (30/125) of participants in the unlikely TB group (OR=7.9, 95% CI=3.9,16.1; *P*<.001). Pericardial effusions (≥0.5 cm) were the most common sonographic finding in those with probable/confirmed TB but were also seen in the unlikely TB group (43% versus 10% respectively, *P*<.001). In the group of participants who had a positive FASH protocol but were not treated for TB, the majority had small pericardial effusions as the only FASH finding. In the probable/confirmed TB group, abdominal lymphadenopathy and pleural effusions were seen in 24% and 14% of patients, respectively, and were rarely seen in the unlikely TB group at 2% and 1%, respectively (*P*<.001). Ascites was also seen more frequently with probable/confirmed TB compared to unlikely TB (16% versus 4% respectively, *P*=.01). Hepatic and splenic lesions were uncommon in both groups (Table 2). In a sensitivity analysis comparing only those with confirmed TB (probable TB excluded) to those with unlikely TB, all associations above remained significant except for ascites (Supplementary Table 2). In a sensitivity analysis recategorizing participants who died without microbiologic confirmation from unlikely TB to probable TB, the association of FASH with probable/confirmed TB was strengthened (Supplementary Table 3).

**TABLE 2.** Baseline FASH Findings in Participants at an Urban Medical Center, Lilongwe, Malawi, Stratified by TB Category (N=181)

	Overall, No. (%) (N=181)	Unlikely TB, No. (%) n=125	Probable/Confirmed TB, No. (%) n=56	*P* Value
Pericardial effusion^a^	36 (19.9)	12 (9.6)	24 (42.9)	<.001
Pleural effusion	9 (5.0)	1 (0.8)	8 (14.3)	<.001
Ascites	14 (7.7)	5 (4.0)	9 (16.1)	.01
Abdominal lymphadenopathy	15 (8.3)	2 (1.6)	13 (23.2)	<.001
Liver lesions	4 (2.2)	3 (2.4)	1 (1.8)	>.99
Splenic lesions	5 (2.8)	2 (1.6)	3 (5.4)	.17
FASH positive^b^	70 (38.7)	30 (24.0)^c^	40 (71.4)	<.001
FASH negative	111 (61.3)	95 (76.0)	16 (28.6)	<.001

Abbreviations: FASH, focused assessment with sonography for HIV-associated tuberculosis; TB, tuberculosis.

a Trace pericardial effusions were excluded from the analysis due to unclear clinical significance.

b Any single finding of the protocol is positive.

c Positive FASH for unlikely TB subgroups: (1) low clinical suspicion with improvement=19/111; (2) low clinical suspicion without improvement= 5/8; (3) High clinical suspicion, treated, without improvement=6/6.

### Predictive Value of the FASH Protocol

Individual FASH findings were highly specific for probable/confirmed TB (≥90%), but not sensitive (range 2%–43%). Abdominal lymphadenopathy, followed by pleural and pericardial effusions, had the highest positive predictive value and positive likelihood ratio for probable/confirmed TB (Table 3). A positive FASH, defined as any single finding of the protocol being positive, raised the sensitivity to 71% while lowering the specificity to 76%.

**TABLE 3.** Associations of FASH Findings With Probable/Confirmed TB in Participants at an Urban Medical Center, Lilongwe, Malawi (N=56)

Variable	OR (95% CI)	Sensitivity (95% CI)	Specificity (95% CI)	PPV (95% CI)	NPV (95% CI)	PLR (95% CI)	NLR (95% CI)
Pericardial effusion^a^	7.06 (3.18, 15.66)	0.43 (0.30, 0.57)	0.90 (0.84, 0.95)	0.67 (0.49, 0.81)	0.78 (0.70, 0.84)	4.30 (2.32, 7.97)	0.63 (0.50, 0.80)
Pleural effusion	8.91 (2.35, 33.82)	0.18 (0.09, 0.30)	0.98 (0.93, 1.00)	0.77 (0.46, 0.95)	0.73 (0.65, 0.79)	9.00 (2.58, 31.45)	0.84 (0.74, 0.95)
Ascites	4.60 (1.47, 14.44)	0.16 (0.08, 0.28)	0.96 (0.91, 0.99)	0.64 (0.35, 0.87)	0.72 (0.64, 0.79)	4.00 (1.40, 11.39)	0.88 (0.78, 0.99)
Abdominal lymphadenopathy	13.35 (3.66, 48.71)	0.25 (0.14, 0.38)	0.98 (0.93, 1.00)	0.82 (0.57, 0.96)	0.74 (0.67, 0.81)	12.50 (3.74, 41.80)	0.77 (0.66, 0.90)
Hepatic lesions	0.74 (0.08, 7.27)	0.02 (0.00, 0.10)	0.98 (0.93, 1.00)	0.25 (0.01, 0.81)	0.69 (0.62, 0.76)	1.00 (0.11,9.40)	1.00 (0.96, 1.05)
Splenic lesions	3.48 (0.57, 21.43)	0.05 (0.01, 0.15)	0.98 (0.94, 1.00)	0.60 (0.15, 0.95)	0.70 (0.63, 0.77)	2.50 (0.43, 14.55)	0.97 (0.91, 1.04)
FASH positive	7.92 (3.89, 16.12)	0.71 (0.58, 0.83)	0.76 (0.68, 0.83)	0.57 (0.45, 0.69)	0.86 (0.78, 0.92)	2.96 (2.08, 4.21)	0.38 (0.25, 0.58)

Abbreviations: CI, confidence interval; FASH, focused assessment with sonography for HIV-associated tuberculosis; NLR, negative likelihood ratio; NPV, negative predictive value; OR, odds ratio; PLR, positive likelihood ratio; PPV, positive predictive value; TB, tuberculosis.

a Trace pericardial effusions were excluded from the analysis due to unclear clinical significance.

### Impact of FASH on Clinical Decision Making

**FIGURE 2:**
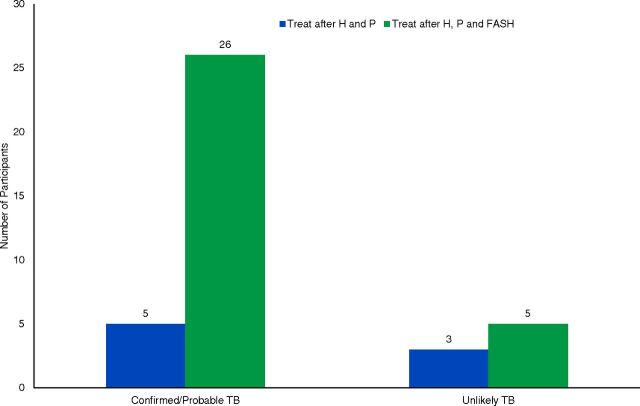
Comparison of Clinicians' Decision to Empirically Treat TB in Participants at 2 Time Points at an Urban Medical Center, Lilongwe, Malawi, by TB Category^a^ Abbreviations: FASH, focused assessment with sonography for HIV-associated tuberculosis; H, history; P, physical exam; TB, tuberculosis. ^a^TB categories determined by study authors after 6-month follow-up.

Clinicians reported that the FASH protocol aided clinical decision making in 76% of encounters. Based on history and physical exam alone, clinicians reported that they would empirically treat 9% (5/56) of participants who were ultimately classified into the probable/confirmed TB group. After the FASH protocol was completed and before any further diagnostic tests were conducted, clinicians reported they would empirically treat 46% (26/56) of these same participants. Of the 36 participants with probable TB (clinically diagnosed and treated, with complete resolution of symptoms), FASH provided sonographic evidence of TB in 75% (27/36) of participants. For those individuals classified as unlikely TB, clinicians reported they would treat 2% (3/125) of participants after the history and physical exam and 4% (5/125) after the FASH protocol and before any further diagnostic tests were conducted (Figure 2).

Clinicians reported that the FASH protocol aided clinical decision making in 76% of encounters.

## DISCUSSION

Our data show the diagnostic utility of the FASH protocol in Malawi and support the recently published 2018 Malawi TB guidelines that recommend the use of FASH to improve the diagnosis of TB in individuals coinfected with HIV.^20^ Our data are consistent with prior studies from other settings that demonstrate that the FASH protocol is predictive of TB.^5^^,^^8^^,^^9^^,^^14^^,^^15^^,^^21^^,^^22^ A recent study from Malawi noted that the majority (61%) of TB diagnoses could not be confirmed microbiologically,^23^ leaving the clinician with a challenging decision: initiate the unconfirmed individual on prolonged and potentially toxic medications, or defer therapy, risking morbidity and mortality from untreated TB. Delays in TB diagnosis in individuals who have HIV result in high morbidity and mortality, so any intervention that can reduce time to TB treatment has the potential to have a significant impact on clinical outcomes.^24^

Our study is unique in that we assessed clinicians' suspicion of TB at 2 time points: (1) after completion of the history and physical exam, and (2) after completion of the FASH protocol, but before any other diagnostic tests were performed, allowing for a real-time assessment of how FASH influenced the decision making of clinicians in settings where chest radiography and lab diagnostics may not be available. This is relevant because in many rural settings in Malawi, traditional radiography and lab diagnostics remain limited while POCUS equipment is becoming increasingly available. Therefore, FASH could help clinicians decide to start empiric TB treatment while awaiting test results and/or referring to facilities for further diagnostic testing. In our study, when the history and physical exam were less consistent with TB, the FASH protocol did not increase the clinician's clinical concern for TB, therefore FASH did not contribute to overtreatment. Among those with probable/confirmed TB, FASH led to a 5-fold increase in the clinician's decision to initiate treatment on that day.

Our study is unique in assessing clinicians' suspicion of TB both after completing a history and physical exam and after completing the FASH protocol.

Among those with probable/confirmed TB, FASH led to a 5-fold increase in the clinician's decision to initiate treatment on that day.

When performing the FASH protocol, if a patient has certain findings, such as abdominal lymphadenopathy, pleural effusions, and splenic lesions, the odds of the patient having TB are high. These findings should warrant strong consideration for empiric treatment.^8^^,^^22^ A recent study evaluating FASH in participants with culture proven HIV-associated TB demonstrated that FASH is more specific than sensitive, and specificity continues to increase with incrementally positive FASH findings.^12^ Conversely, FASH should not be used to exclude a diagnosis of TB given its low sensitivity. It is important to consider other etiologies that may lead to positive FASH findings, such as Kaposi sarcoma, lymphoma, and non-TB mycobacteria. It is also possible that untreated HIV can be associated with findings, such as abdominal lymphadenopathy, without any other contributory coinfection. However, prior studies demonstrate that lymphadenopathy ≥1.0–1.5 cm is characteristic of TB with HIV.^7^^,^^11^^,^^12^ It is also important to note that the cause of positive FASH findings differ in individuals who do not have HIV or in environments with low TB prevalence.^8^

Contrary to prior studies, we had very few participants with splenic lesions, which have been shown to be common and significantly associated with TB.^8^^–^^11^^,^^22^^,^^25^^,^^26^ This is likely secondary to a higher degree of difficulty in acquiring this image and increased sensitivity when using the high frequency linear probe in place of the abdominal curvilinear probe, which was not used in our study.^27^ As FASH training expands across Malawi and similar resource-limited settings, ample time should be dedicated to training on how to obtain splenic images.

### Limitations

Our study has several limitations. First, it is possible that participants in the unlikely or probable TB categories were misclassified due to the lack of microbiological or histological confirmation. This is a common limitation for TB studies in low-resource areas and represents the reality faced by clinicians. This study took a conservative approach and required a high burden of evidence to classify a participant as probable TB. We chose to classify deaths without TB diagnostic confirmation as unlikely TB to avoid the possibility of biasing the results in favor of the FASH protocol. However, many of these individuals presented late, had positive FASH findings, and may have died from TB. We performed a sensitivity analysis recategorizing those who died as probable TB, and our results were strengthened. Second, given our small sample size, we did not exclude participants with pulmonary TB from the sample nor stratify by pulmonary versus extrapulmonary TB. In settings where pulmonary TB can be confirmed at the point-of-care by sputum microscopy and chest radiography, FASH would not be required as an adjunctive tool. Lastly, performing ultrasound has a high degree of interoperator variability. Our study had strong support from an expert radiologist from UCLA who provided continuous and real-time quality assurance. This type of support may not be available for many programs. Expansion of teleradiology to support the implementation of FASH should be considered within resource-limited settings.^18^^,^^28^

## CONCLUSION

The FASH protocol is an adjunctive diagnostic tool that is predictive of TB in individuals with HIV and can augment clinicians' ability to diagnose TB at the point-of-care and reduce delays in treatment, particularly in settings with limited lab diagnostic capacity. The FASH protocol also plays an important role for clinicians who remain concerned for TB coinfection in individuals with an initial negative work-up but ongoing high clinical suspicion. Efforts to evaluate real-world implementation of FASH across a variety of settings will be important for understanding how to best scale this strategy in a range of resource-limited settings.

## Supplementary Material

19-00251-Kahn-Supplementary_Table2.pdf

19-00251-Kahn-Supplementary_Table1.pdf

19-00251-Kahn-Supplementary_Table3.pdf

## Notes

**Peer Reviewed**

**First Published Online:** February 10, 2020
