# Supplementary material for: Diagnostic Utility and Impact on Clinical Decision Making of Focused Assessment With Sonography for HIV-Associated Tuberculosis in Malawi: A Prospective Cohort Study
Source: Glob Health Sci Pract. 2020 Mar 30;8(1):28–37. doi: 10.9745/GHSP-D-19-00251 (PMC7108937; doi:10.9745/GHSP-D-19-00251)
Supplement: 19-00251-Kahn-Supplementary_Table2.pdf [file 19-00251-Kahn-Supplementary_Table2.pdf]

Supplement to: Kahn D, Pool KL, Phiri L, et al. Diagnostic utility and impact on clinical decision making of focused assessment with sonography for HIV-associated tuberculosis in Malawi: a prospective cohort study. *Glob Health Sci Pract*. 2020;8(1). https://doi.org/10.9745/GHSP-D-19-00251

**Supplementary Table 2. FASH Findings in Participants at an Urban Medical Center, Lilongwe, Malawi, Stratified by Unlikely TB and Confirmed TB (Probable TB Excluded) (N=145)**

| | Overall, No. (%) (N=145) | Unlikely TB, No. (%) n=125 | Confirmed TB, No. (%) n=20 | *P* Value |
|---|---|---|---|---|
| Pericardial effusion[a] | 21.0 (14.5) | 12.0 (9.6) | 9.0 (45.0) | <.001 |
| Pleural effusion | 5.0 (3.4) | 1.0 (0.8) | 4.0 (20.0) | <.001 |
| Ascites | 6.0 (4.1) | 5.0 (4.0) | 1.0 (5.0) | >.99 |
| Abdominal lymphadenopathy | 10.0 (6.9) | 2.0 (1.6) | 8.0 (40.0) | <.001 |
| Liver lesions | 4.0 (2.7) | 3.0 (2.4) | 1.0 (5.0) | .45 |
| Splenic lesions | 3.0 (2.1) | 2.0 (1.6) | 1.0 (5.0) | .36 |
| FASH positive[b] | 43.0 (29.7) | 30.0 (24.0) | 13.0 (65.0) | |
| FASH negative | 102.0 (70.3) | 95.0 (76.0) | 7.0 (35.0) | |

Abbreviations: FASH, focused assessment with sonography for HIV-associated tuberculosis; TB, tuberculosis.
[a] Trace pericardial effusions were excluded from the analysis due to unclear clinical significance.
[b] Any single finding of the protocol is positive.
