# Supplementary material for: Diagnostic Utility and Impact on Clinical Decision Making of Focused Assessment With Sonography for HIV-Associated Tuberculosis in Malawi: A Prospective Cohort Study
Source: Glob Health Sci Pract. 2020 Mar 30;8(1):28–37. doi: 10.9745/GHSP-D-19-00251 (PMC7108937; doi:10.9745/GHSP-D-19-00251)
Supplement: 19-00251-Kahn-Supplementary_Table1.pdf [file 19-00251-Kahn-Supplementary_Table1.pdf]

Supplement to: Kahn D, Pool KL, Phiri L, et al. Diagnostic utility and impact on clinical decision making of focused assessment with sonography for HIV-associated tuberculosis in Malawi: a prospective cohort study. *Glob Health Sci Pract*. 2020;8(1). https://doi.org/10.9745/GHSP-D-19-00251

Supplementary Table 1. Baseline Demographic and Clinical Characteristics of Participants at an Urban Medical Center, Lilongwe, Malawi, With Probable and Confirmed TB, Lilongwe, Malawi (N=56)

	**Probable TB (n=36)**	**Confirmed TB (n=20)**	***P* Value**
**Age**, years, median (IQR)	41.0 (34.0–43.0)	37.0 (31.0–42.0)	.24
**Gender**			>.99
Male, No. (%)	19.0 (52.8)	10 (50.0)	
Female, No. (%)	17.0 (47.2)	10 (50.0)	
**Baseline CD4 Count**[a] (cells/mm^3), median (IQR)	126.0 (28.0–171.0)	83 (49.0–182.0)	.91
**Viral Load** (copies/ml)[b]			.60
<1,000, No. (%)	16.0 (44.4)	12 (60.0)	
1,000–50,000, No. (%)	7.0 (19.4)	4 (20.0)	
>50,000, No. (%)	7.0 (19.4)	2 (10.0)	
**Baseline ART Regimen**[c]			.59
TDF/3TC/EFZ, No. (%)	12.0 (33.3)	8 (40.0)	
Other NNRTI-based regimen, No. (%)	2.0 (5.6)	0 (0.0)	
Protease inhibitor-based regimen, No. (%)	1.0 (2.8)	2 (10.0)	
No ART, No. (%)	17.0 (47.2)	9 (45.0)	
**Previous TB**,[d] No. (%)	8.0 (22.2)	4 (20.0)	>.99
**TB Sign/Symptom**[e]			
Fever, No. (%)	25.0 (69.4)	17 (85.0)	.33
Cough, No. (%)	33.0 (91.7)	18 (90.0)	>.99
Night sweats, No. (%)	26.0 (72.2)	11 (55.0)	.24
Weight loss, No. (%)	31.0 (86.1)	19 (95.0)	.41
**BMI**, median (IQR)	18.6 (16.7–20.0)	17.8 (16.5–19.1)	.27

Abbreviations: ART, antiretroviral therapy; BMI, body mass index; IQR, interquartile range; NNRTI, non-nucleoside reverse transcriptase inhibitor; TB, tuberculosis; TDF/3TC/EFZ, tenofovir disoproxil fumarate/lamivudine/efavirenz.

[a] Missing in 1 individual.

[b] Missing in 8 individuals.

[c] Missing in 5 individuals.

[d] Based on participant self report.

[e] Participants could have 1 or more signs/symptoms based on screening questions at study entry.
