# Supplementary material for: Diagnostic Utility and Impact on Clinical Decision Making of Focused Assessment With Sonography for HIV-Associated Tuberculosis in Malawi: A Prospective Cohort Study
Source: Glob Health Sci Pract. 2020 Mar 30;8(1):28–37. doi: 10.9745/GHSP-D-19-00251 (PMC7108937; doi:10.9745/GHSP-D-19-00251)
Supplement: 19-00251-Kahn-Supplementary_Table3.pdf [file 19-00251-Kahn-Supplementary_Table3.pdf]

Supplement to: Kahn D, Pool KL, Phiri L, et al. Diagnostic utility and impact on clinical decision making of focused assessment with sonography for HIV-associated tuberculosis in Malawi: a prospective cohort study. *Glob Health Sci Pract*. 2020;8(1). https://doi.org/10.9745/GHSP-D-19-00251

Supplementary Table 3. FASH Findings of Participants at an Urban Medical Center, Lilongwe, Malawi, Who Died Without Microbiologic Confirmation (n=12) Reclassified From Unlikely TB Group Into Probable/Confirmed TB Group, Stratified by TB Status

	Overall, No. (%) N=181	Unlikely TB, No. (%) n=113	Probable/Confirmed TB, No. (%) n=68	*P* Value
Pericardial effusion[a]	36 (19.9)	6 (5.3)	30 (44.1)	<.001
Pleural effusion	9 (5.0)	1 (0.9)	8 (11.8)	<.001
Ascites	14 (7.7)	3 (2.7)	11 (16.2)	.002
Abdominal lymphadenopathy	15 (8.3)	0 (0)	15 (22.4)	<.001
Liver lesions	4 (2.2)	2 (1.8)	2 (2.9)	.63
Splenic lesions	5 (2.8)	2 (1.8)	3 (4.4)	.37
FASH positive[b]	70 (38.7)	20 (17.7)	50 (73.5)	<.001
FASH negative	111 (61.3)	93 (82.3)	18 (26.5)	<.001

Abbreviations: FASH, focused assessment with sonography for HIV-associated tuberculosis; TB, tuberculosis.
[a] Trace pericardial effusions were excluded from the analysis due to unclear clinical significance.
[b] Any single finding of the protocol is positive.
